# Role of *TNFSF15* variants in oral cancer development and clinicopathologic characteristics

**DOI:** 10.1111/jcmm.17569

**Published:** 2022-10-13

**Authors:** Hsueh‐Ju Lu, Chun‐Yi Chuang, Chun‐Wen Su, Mu‐Kuan Chen, Wei‐En Yang, Chia‐Ming Yeh, Chih‐Hsin Tang, Chiao‐Wen Lin, Shun‐Fa Yang

**Affiliations:** ^1^ Division of Hematology and Oncology, Department of Internal Medicine Chung Shan Medical University Hospital Taichung Taiwan; ^2^ School of Medicine Chung Shan Medical University Taichung Taiwan; ^3^ Department of Otolaryngology Chung Shan Medical University Hospital Taichung Taiwan; ^4^ Department of Medical Research Chung Shan Medical University Hospital Taichung Taiwan; ^5^ Institute of Medicine Chung Shan Medical University Taichung Taiwan; ^6^ Department of Otorhinolaryngology‐Head and Neck Surgery Changhua Christian Hospital Changhua Taiwan; ^7^ Oral cancer Research Center Changhua Christian Hospital Changhua Taiwan; ^8^ School of Medicine China Medical University Taichung Taiwan; ^9^ Chinese Medicine Research Center China Medical University Taichung Taiwan; ^10^ Department of Medical Laboratory Science and Biotechnology College of Medical and Health Science, Asia University Taichung Taiwan; ^11^ Institute of Oral Sciences Chung Shan Medical University Taichung Taiwan; ^12^ Department of Dentistry Chung Shan Medical University Hospital Taichung Taiwan

**Keywords:** oral cavity squamous cell carcinoma, polymorphism, survival, TNFSF15

## Abstract

Tumour necrosis family superfamily (TNFSF) member 15 (TNFSF15), encoded by *TNFSF15*, regulates immune responses and inflammation. However, the roles of *TNFSF15* single‐nucleotide variants (SNVs; formerly SNPs) in oral cavity squamous cell carcinoma (OCSCC) remain unclear. This case–control study included 2523 participants (1324 patients with OCSCC [52.5%] and 1199 healthy controls [47.5%]). The effects of *TNFSF15* rs3810936, rs6478108 and rs6478109 on cancer development and prognosis were analysed by real‐time PCR genotype assay. The Genotype‐Tissue Expression (GTEx) and The Cancer Genome Atlas (TCGA) databases were used to validate our findings. The results demonstrated that the patients with altered *TNFSF15* SNVs had poorer histological differentiation than did those with wild‐type alleles. *TNFSF15* SNVs were significantly associated with moderate‐to‐poor histological differentiation in univariate logistic regression. In the GTEx database, the expression of altered *TNFSF15* SNVs in whole blood was lower than that of wild‐type alleles. However, the expression of altered SNVs in the upper aerodigestive mucosa was higher than that of wild‐type alleles. In the TCGA database, the patients with higher TNFSF15 expression had shorter overall survival than did those with lower TNFSF15 expression, especially for human papillomavirus‐negative and advanced staging groups. In conclusion, although *TNFSF15* SNVs did not affect OCSCC development, the patients with altered *TNFSF15* SNVs exhibited poorer histological differentiation. The patients with higher TNFSF15 expression had poorer prognosis than did those with lower TNFSF15 expression.

## 1 INTRODUCTION

Oral cavity squamous cell carcinoma (OCSCC) is the largest subgroup of head and neck squamous cell carcinoma (HNSCC), which is the seventh most common cancer globally and the fourth most common cancer in men in Taiwan.^1^, ^2^, ^3^ However, up to 50% of patients with OCSCC experience local recurrence or distant metastasis after curative surgery,^4^, ^5^, ^6^ and the median overall survival (OS) of patients with recurrent metastatic OCSCC was only 12–14 months.^7^, ^8^, ^9^ Because of the poor prognosis of patients with OCSCC, the identification of biomarkers predicting cancer development and prognosis is crucial.

The tumour necrosis factor (TNF) superfamily includes 19 ligands and 30 receptors.^10^ TNF superfamily member 15 (TNFSF15), also named TNF‐like ligand 1A (TL1A), is a ligand encoded by *TNFSF15* that is mapped on chromosome 9q32. Death receptor 3 (DR3) is the main receptor of TNFSF15.^11^ In addition to coactivating T cells and stimulating dendritic cell maturation, some studies reported that in the tumour, TNFSF15 might promote lymphatic metastasis through assisting lymphangiogenesis. TNFSF15 was associated with carcinogenesis and poor prognosis.^12^, ^13^, ^14^ Several studies have reported that *TNFSF15* single‐nucleotide variations (SNVs; formerly SNPs) are associated with the development of inflammatory bowel disease (IBD).^15^, ^16^, ^17^ In addition, many studies have reported the roles of *TNFSF15* SNVs in cancer development.^13^, ^18^ However, the effects of *TNFSF15* SNVs in OCSCC remain unclear.

The development of OCSCC is associated with the formation of clinical precancerous lesions including leukoplakia and erythroplakia.^19^, ^20^ Habits such as tobacco smoking, alcohol drinking and betel quid chewing have been reported to substantially accelerate the development of these precancerous lesions.^21^, ^22^, ^23^ The mechanisms which lead to precancerous lesions and the formation of OCSCC are complex. Ali et al. study reported these personal habits were associated with several genetic variations, including tumour suppressor genes, proto‐oncogenes, oncogenes and genes controlling normal cellular processes.^24^ Others, including genotoxicity, reactive oxygen species (ROS), accumulation of DNA damage and clonal selection, were also reported to be related to these personal habits.^25^, ^26^, ^27^, ^28^, ^29^ In addition, one of the most important is that these habits lead to tissue inflammation,^30^ and the inflammatory changes result in the development of OCSCC and worsen the prognosis of patients with OCSCC..^21^, ^31^, ^32^ For example, the major component of betel quid is betel nut, which contains areca alkaloids including arecoline, arecaidine, guvacoline and guvacine.^33^, ^34^ And ROS, one of the production from cellular metabolism of betel quid, also causes preneoplastic alterations and the formation of OCSCC.^35^ These components trigger proinflammatory cytokine secretion and increase cell proliferation, thus causing the development of inflammatory disorders and OCSCC in betel quid chewers.^36^

TNFSF15 regulates both innate and adaptive immune cells.^37^ And TNFSF15‐associated DR3 signalling was critical for enhancing MAPK/NF‐κB/PI3K signalling and cytokine secretion in macrophages.^38^, ^39^ The signalling was related to the proinflammatory pathway, proliferative pathway, and cell death pathways.^39^ TNFSF15 SNVs, such as rs3810936, rs6478108 and rs6478109, have also been reported to be significantly associated with the development of inflammatory diseases and increasing cancer development.^13^, ^40^, ^41^, ^42^ Although TNFSF15 was significantly related to tissue inflammation and carcinogenesis, the interaction between TNFSF15, tissue inflammation, and cancer development in OCSCC was unknown.

This study examined the role of *TNFSF15* SNVs in the development and prognosis of OCSCC by retrospectively enrolling patients with OCSCC and healthy controls. All the participants underwent testing for *TNFSF15* SNVs. Bioinformatics databases, namely the Genotype‐Tissue Expression (GTEx) Portal and *The Cancer Genome Atlas* (*TCGA*), were used to validate our results. The findings of this study provide insights into the effect of *TNFSF15* SNVs on OCSCC development.

## 2 MATERIALS AND METHODS

### 2.1 Study participants

In this case–control study, we retrospectively enrolled patients who received a pathological diagnosis of OCSCC between 2007 and 2019 at Chung Shan Medical University Hospital and Changhua Christian Hospital and included them in the case group. Patients without pathologic diagnosis, and those with second primary malignancies were excluded. In addition, healthy participants aged between 30 and 70 years with normal mental capacity and no cancer history were enrolled in the control group from the Taiwan Biobank. Because approximately 90% of patients with OCSCC were men, female participants were excluded from both the case and control groups. This study was approved by the Institutional Review Board of Chung Shan Medical University Hospital (CSMUH No: CS15125).

Details regarding the following basic characteristics of the case and control groups were obtained from the Biobank databases: age, cigarette smoking, alcohol drinking and betel quid chewing. Clinical staging and histological differentiation were provided for the case group only. The seventh edition of the American Joint Committee on Cancer staging system was used in this study.^43^ Because of delinking and anonymity, we could not retrospectively record clinical outcomes in this study.

### 2.2 DNA extraction and genotyping

Whole‐blood specimens were collected and placed in sterile tubes containing ethylenediaminetetraacetic acid. These specimens were immediately centrifuged and then stored at −80°C. Genomic DNA was extracted from peripheral blood leukocytes by using QIAamp DNA blood mini kits (Qiagen, Valencia, CA) according to previously described.^44^, ^45^ Genomic DNA was dissolved in TE buffer (10 mM trisaminomethane and 1 mM ethylenediaminetetraacetic acid; pH 7.8) and then quantified by measuring the optical density at 260 nm. The final product was stored at −20°C and used as a template for polymerase chain reaction. *TNFSF15* rs3810936, rs6478108, and rs6478109 have been reported to be significantly associated with the development of inflammatory diseases and cancer.^13^, ^40^, ^41^ Therefore, we chose these candidate loci in our study. The results were analysed using SDS version 3.0. Details regarding DNA extraction and genotyping were published in our previous study.^46^, ^47^

### 2.3 Published databases for validation

Published databases, namely dbSNP, the GTEx portal and cBioPortal, were used to validate our results. dbSNP contains details regarding human SNVs, microsatellites, and small‐scale insertions and deletions along with publication, population frequency, molecular consequence and genomic and RefSeq mapping information for both common and clinical variations (www.ncbi.nlm.nih.gov/snp/).^48^ The GTEx portal, a comprehensive public resource used to study tissue‐specific gene expression and regulation, provides open‐access data on gene expression, quantitative trait loci (QTLs), and histology images from the 54 nondiseased tissue sites of approximately 1000 individuals (gtexportal.org/home/).^49^ The TCGA database was downloaded from cBioPortal, an open‐source software system used to visualize variant and gene expression data from TCGA (www.cbioportal.org/).^50^, ^51^

### 2.4 Statistical analysis

Clinicopathological parameters were compared using the *χ* ^2^ test and Fisher's exact test. The Mann–Whitney U test was used for continuous variables. Odds ratios (ORs) for cancer development and histological differentiation were calculated by performing univariate and multivariate logistic regression analyses. To investigate the effect of *TNFSF15* SNVs on OCSCC development, we calculated adjusted ORs (AORs) after adjustment for personal habits and age because personal habits significantly affect the development of OCSCC.^21^ We performed the log‐rank test and used Kaplan–Meier plots to analyse survival. A two‐sided *p* < 0.05 was considered statistically significant. All statistical analyses were performed using SPSS (version 21.0, SPSS Inc., Chicago, IL).

## 3 RESULTS

### 3.1 Baseline characteristics

This study recruited 2523 participants, of whom 1324 (52.5%) were included in the case group and 1199 (47.5%) in the control group. No difference in age was noted between the groups. However, a significantly higher proportion of the participants in the case group smoked cigarettes, consumed alcohol and chewed betel quid than did those in the control group (all *p* < 0.001). Table 1 lists the basic characteristics of the participants.

**TABLE 1:** Basic characteristics of healthy control and patients with oral cancer

Variable	Patients (*N* = 1324)	Controls (*N* = 1199)	*p* value
Age (yrs)			
≥55	705 (53.3)	633 (52.8)	0.425
<55	619 (46.8)	566 (47.2)	
Cigarette smoking			
Yes	1115 (84.2)	636 (53.0)	<0.001
No	209 (15.8)	563 (47.0)	
Alcohol drinking			
Yes	625 (47.2)	237 (19.8)	<0.001
No	699 (52.8)	962 (80.2)	
Betel quid chewing			
Yes	989 (74.7)	199 (16.6)	<0.001
No	335 (25.3)	1000 (83.4)	
Clinical staging			
I + II	623 (47.1)		
III + IV	701 (52.9)		
Clinical T staging			
T1 + T2	667 (50.4)		
T3 + T4	657 (49.6)		
Clinical N staging			
N0	871 (65.8)		
N+	453 (34.2)		
Clinical M staging			
M0	1314 (99.2)		
M1	10 (0.8)		
Histological differentiation			
Well	185 (14.0)		
Moderate to poor	1139 (86.0)		

### 3.2 TNFSF15 SNVs

*TNFSF15* rs3810936, rs6478108 and rs6478109 are all located on chromosome 9 and were examined in all the participants. According to the 1000 Genomes Project, the allele frequencies of these three SNVs were 50.7%, 51.0% and 51.2% for the East Asian population, respectively. Based on Clinvar, the clinical significance of these SNVs was unclear (Table S1).

### 3.3 *TNFSF15* SNVs did not affect the development of OCSCC

The distributions and ORs between the case and control groups are presented in Table 2. In the control group, the genotypic frequencies of *TNFSF15* rs3810936, rs6478108 and rs6478109 were in Hardy–Weinberg equilibrium (*p* > 0.05). The allelic variant frequencies of *TNFSF15* rs3810936, rs6478108 and rs6478109 were 69.7% (1758/2523), 73.4% (1851/2523) and 73.8% (1861/2523) for all the participants and 69.9% (830/1188), 72.8% (865/1188) and 73.4% (872/1188) for the betel quid chewers, respectively. The distributions of allelic variants did not differ between the case and control groups (*p* = 0.850, 0.821 and 0.960 for all the participants and *p* = 0.972, 0.697 and 0.753 for betel quid chewers, respectively, for rs3810936, rs6478108 and rs6478109). To investigate the effect of *TNFSF15* SNVs on OCSCC development, the ORs and AORs of these three SNVs were calculated. The results revealed that the allelic variants did not affect the development of OCSCC in all the participants or betel quid chewers.

**TABLE 2:** Odds ratios (OR) and 95% *confidence interval* (*CI)* of oral cancer associated with *TNFSF15* genotypic frequencies

Variable	Patients (*N*, %)	Controls (*N*, %)		OR (95% CI)	AOR (95% CI)^a^
All participants					
	*N* = 1324	*N* = 1199	*p* value		
rs3810936					
TT	398 (30.1)	367 (30.6)	0.850	1.000 (reference)	1.000 (reference)
TC	657 (49.6)	599 (50.0)		1.011 (0.845–1.211)	1.006 (0.805–1.259)
CC	269 (20.3)	233 (19.4)		1.065 (0.850–1.334)	1.011 (0.764–1.339)
TC + CC	926 (69.9)	832 (69.4)		1.026 (0.866–1.216)	1.008 (0.816–1.244)
rs6478108					
CC	358 (27.0)	314 (26.2)	0.821	1.000 (reference)	1.000 (reference)
CT	672 (50.8)	608 (50.7)		0.969 (0.804–1.169)	0.998 (0.791–1.258)
TT	294 (22.2)	277 (23.1)		0.931 (0.745–1.164)	0.898 (0.678–1.188)
CT + TT	966 (73.0)	885 (73.8)		0.957 (0.802–1.143)	0.967 (0.776–1.203)
rs6478109					
AA	349 (26.4)	313 (26.1)	0.960	1.000 (reference)	1.000(reference)
AG	672 (50.8)	606 (50.5)		0.995 (0.824–1.200)	1.015 (0.803–1.282)
GG	303 (22.9)	280 (23.4)		0.971 (0.777–1.213)	0.951 (0.719–1.258)
AG + GG	975 (73.6)	886 (73.9)		0.987 (0.826–1.179)	0.995 (0.798–1.240)

Betel quid chewer					
	*N* = 989	*N* = 199			
rs3810936					
TT	299 (30.2)	59 (29.6)	0.972	1.000 (reference)	1.000 (reference)
TC	488 (49.3)	98 (49.2)		0.983 (0.690–1.399)	0.988 (0.693–1.410)
CC	202 (20.4)	42 (21.1)		0.949 (0.615–1.465)	0.934 (0.602–1.450)
TC + CC	690 (69.8)	140 (70.3)		0.973 (0.697–1.357)	0.977 (0.699–1.365)
rs6478108					
CC	273 (27.6)	50 (25.1)	0.697	1.000 (reference)	1.000 (reference)
CT	494 (49.9)	100 (50.3)		0.905 (0.625–1.310)	0.909 (0.626–1.319)
TT	222 (22.4)	49 (24.6)		0.830 (0.539–1.278)	0.816 (0.528–1.262)
CT + TT	716 (72.8)	149 (74.9)		0.880 (0.621–1.248)	0.882 (0.621–1.253)
rs6478109					
AA	267 (27.0)	49 (24.6)	0.753	1.000 (reference)	1.000 (reference)
AG	495 (50.1)	101 (50.8)		0.899 (0.620–1.305)	0.899 (0.618–1.309)
GG	227 (23.0)	49 (24.6)		0.850 (0.551–1.312)	0.843 (0.544–1.306)
AG + GG	722 (73.0)	150 (75.4)		0.883 (0.621–1.256)	0.886 (0.622–1.263)

^a^ Adjusted for the effects of age, cigarette smoking, alcohol drinking and betel quid chewing.

### 3.4 Prognostic role of *TNFSF15* SNVs in OCSCC

We examined the prognostic role of altered *TNFSF15* SNVs in OCSCC. In the case group, those with altered *TNFSF15* SNVs had poorer histological differentiation than did those with wild‐type *TNFSF15* SNVs (rs3810936, *p* = 0.009; rs6478108, *p* = 0.014 and rs6478109, *p* = 0.008) (Table 3). Furthermore, in the subgroups of patients who smoked cigarettes, consumed alcohol, and chewed betel quid, those with altered *TNFSF15* SNVs had poorer histological differentiation than did those with wild‐type *TNFSF15* SNVs (Tables S2, S3 and S4).

**TABLE 3:** Distributions of demographical characteristics of *TNFSF15* allele mutation in all OCSCC patients (*N* = 1324)

	rs3810936			rs6478108			rs6478109		
Variable	TC + CC	TT	*p* value	CT + TT	CC	*p* value	AG + GG	AA	*p* value
	(*N* = 926)	*N* = 398)		(*N* = 966)	(*N* = 358)		(*N* = 975)	(*N* = 349)	
Age > = 55	503 (54.3)	202 (50.8)	0.129	525 (54.3)	180 (50.3)	0.103	532 (54.6)	174 (49.6)	0.062
Personal history									
cigarette smoking	784 (84.7)	331 (83.2)	0.271 0.124	815 (84.4)	300 (83.8)	0.430	823 (84.4)	292 (83.7)	0.401
alcohol drinking	427 (46.1)	198 (49.7)	0.124	449 (46.5)	176 (49.2)	0.210	452 (46.4)	173 (49.6)	0.166
betel quid chewing	690 (74.5)	299 (75.1)	0.436	716 (74.1)	273 (76.3)	0.236	722 (74.1)	267 (76.5)	0.203
Clinical staging			0.227			0.159			0.099
Stage I + II	429 (46.3)	194 (48.7)							
Stage III + IV	497 (53.7)	204 (51.3)		520 (53.8)	181 (50.6)		527 (54.1)	174 (49.9)	
Clinical T staging			0.360			0.188			0.202
T1/2	463 (50.0)	204 (51.3)		479 (49.6)	188 (52.5)		484 (49.6)	183 (52.4)	
T3/4	463 (50.0)	194 (48.7)		487 (50.4)	170 (47.5)		491 (50.4)	166 (47.6)	
Clinical N staging			0.200			0.096			0.044
N0	602 (65.0)	269 (67.6)		625 (64.7)	246 (68.7)		628 (64.4)	243 (69.6)	
N+	324 (35.0)	129 (32.4)		341 (35.3)	112 (31.3)		347 (35.6)	106 (30.4)	
Metastasis			0.352			0.535			0.515
M0	920 (99.0)	394 (99.0)		959 (99.3)	355 (99.2)		968 (99.3)	346 (99.1)	
M1	6(0.6)	4 (1.0)		7 (0.7)	3(0.8)		7 (0.7)	3(0.9)	
Cell differentiated grade			0.009			0.014			0.008
Well	115 (12.4)	70 (17.6)		122 (12.6)			122 (12.5)	63 (18.1)	
Moderate or poor	811 (87.4)	328 (82.4)		844 (87.4)	295 (82.4)		853 (87.5)	286 (81.9)	

In the univariate logistic regression analysis, altered *TNFSF15* SNVs were significantly associated with moderate‐to‐poor histological differentiation in all the participants (rs3810936, OR [95% confidence interval] = 1.505 [1.089–2.080], *p* = 0.013; rs6478108, 1.477 [1.060–2.059], *p* = 0.021; and rs6478109, 1.540 [1.105–2.147], *p* = 0.011). For the betel quid chewers, *TNFSF15* SNVs were crucial for histological differentiation (rs3810936, 1.753 [1.224–2.512], *p* = 0.002; rs6478108, 1.729 [1.199–2.492], *p* = 0.003 and rs6478109, 1.795 [1.244–2.589], *p* = 0.002; Table 4).

**TABLE 4:** Univariate and multivariate logistic regression for moderate to poor histologic differentiation in all oral cancer patients

	All patients		Betel quid chewer	
	Univariate	Multivariate	Univariate	Multivariate
Variable	OR (95% CI), *p* value	OR (95% CI), *p* value	OR (95% CI), *p* value	OR (95% CI), *p* value
Age (yrs)				
≥55 vs. <55	0.870 (0.636–1.190), 0.383		0.883 (0.608–1.223), 0.406	
Personal history				
cigarette smoking (yes vs. no)	0.609 (0.373–0.994), 0.047	0.681 (0.395–1.176), 0.168	0.477 (0.188–1.210), 0.119	
alcohol drinking (yes vs. no)	0.983 (0.720–1.342), 0.915		0.969 (0.682–1.376), 0.860	
betel quid chewing (yes vs. no)	0.679 (0.679–1.000), 0.050	0.808 (0.395‐1.176), 0.335		
Clinical T staging				
T3/4 vs. T1/2	1.020 (0.748–1.392), 0.899		0.771 (0.543–1.093), 0.144	
Clinical N staging				
N+ vs. N0	2.485 (1.687–3.659), <0.001	2.413 (1.635–3.560), <0.001	2.299 (1.496–3.532), <0.001	2.238 (1.453–3.448), <0.001
Metastasis				
M1 vs. M0	0.647 (0.136–3.072), 0.584		0.440 (0.085–2.290), 0.329	
rs3810936				
TC + CC vs. TT	1.505 (1.089–2.080), 0.013	1.354 (0.863–2.125), 0.188	1.753 (1.224–2.512), 0.002	1.476 (0.892–2.443), 0.130
rs6478108				
CT + TT vs. CC	1.477 (1.060–2.059), 0.021	0.304 (0.026–3.527), 0.341	1.729 (1.199–2.492), 0.003	0.379 (0.028–5.041), 0.462
rs6478109				
AG + GG vs. AA	1.540 (1.105–2.147), 0.011	3.913 (0.346–44.240), 0.270	1.795 (1.244–2.589), 0.002	3.455 (0.267–44.634), 0.342

### 3.5 TNFSF15 mRNA expression varies among different tissues

**FIGURE 1:**
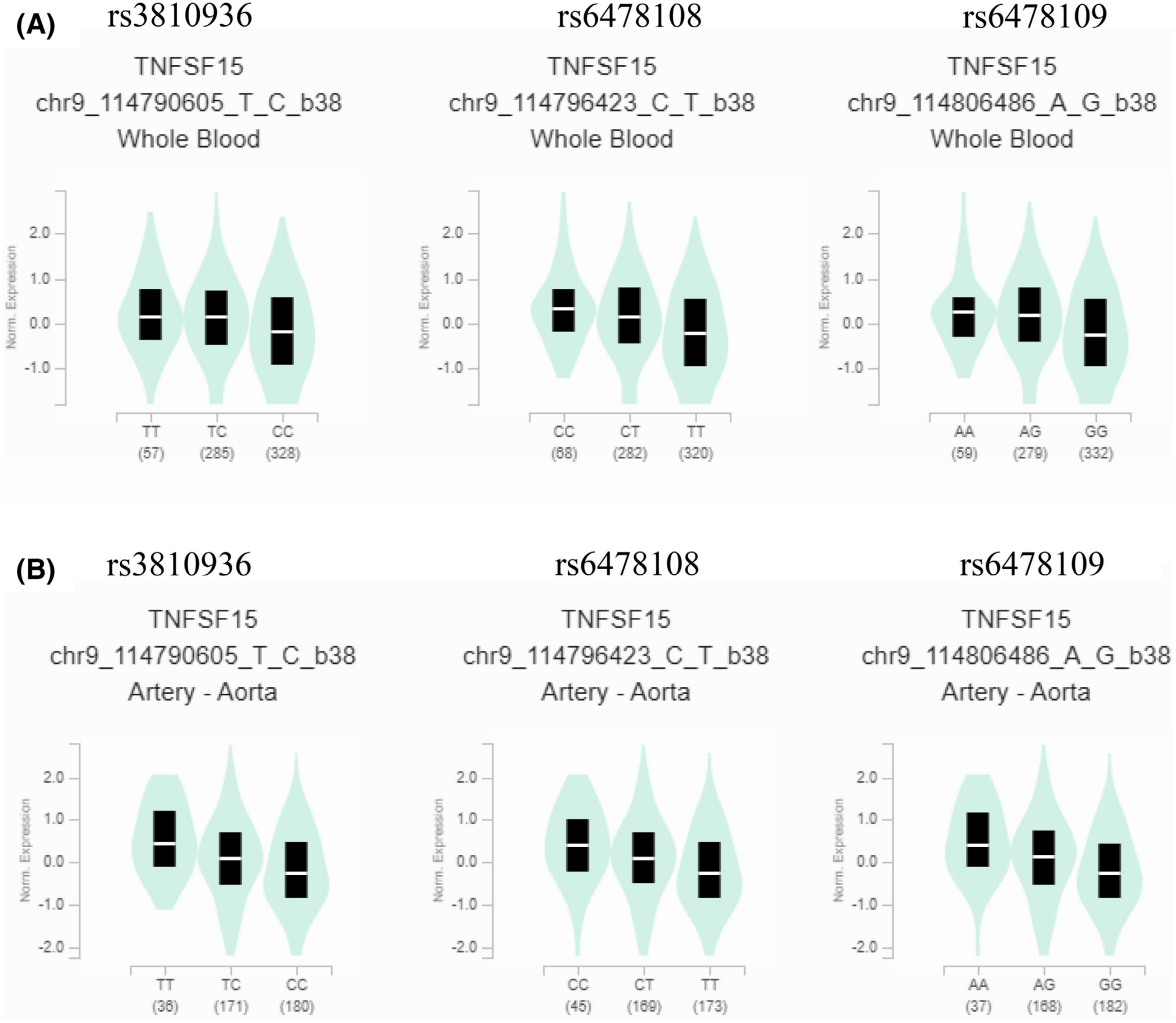
Validated results of TNFSF15 expression by Genotype‐Tissue Expression (GTEx) Portal (https://www.gtexportal.org/home/). In GTEx, violin plots of *TNFSF15* rs3810936, rs6478108 and rs6478109 mutation was associated with lower TNFSF15 expression level in (A) whole blood and (B) artery system than those of *TNFSF15* allele normal type (All *p* < 0.001)

Published bioinformatics databases were used to validate our results. In the GTEx database, the expression of the altered alleles of *TNFSF15* rs3810936, rs6478108 and rs6478109 was significantly lower than that of the wild‐type alleles of *TNFSF15* SNVs in both whole blood and artery‐aorta (all *p* < 0.001; Figure 1 and Figure S1). By contrast, the multitissue expression of QTLs indicated that the expression of altered alleles was higher than that of wild‐type alleles in the upper aerodigestive (oesophagus) mucosa. The single‐tissue QTL normalized effect size and *p* value of *TNFSF15* rs3810936 were 0.0791 and 0.01 for the upper aerodigestive (oesophagus) mucosa and −0.250 and <0.01 for whole blood, respectively (Figure S1A). In addition, *TNFSF15* rs6478108 and rs6478109 exhibited the same expression in the upper aerodigestive (oesophagus) mucosa and whole blood (Figure S1B, C). In summary, the expression of altered *TNFSF15* alleles was lower than that of wild‐type alleles in whole blood; however, the expression was opposite in the upper aerodigestive (oesophagus) mucosa.

### 3.6 Relationship between TNFSF15 expression and clinical outcomes

**FIGURE 2:**
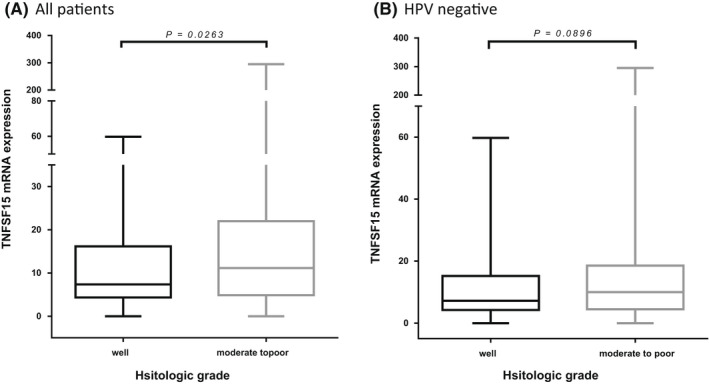
Results of TNFSF15 expression In TCGA database. In TCGA database, patients with moderate to poor histologic differentiation were higher TNFSF15 expression than those with well differentiation, both in (A) all OCSCC and (B) HPV negative population

We used the TCGA database to validate our results. Because two‐thirds of our population had altered *TNFSF15* alleles and the expression of altered *TNFSF15* alleles in the upper aerodigestive (oesophagus) mucosa was higher than that of normal alleles, 515 patients with HNSCC from the TCGA database were divided into high (66.6%, 353/514) and low (33.4%, 172/515) TNFSF15 expression groups based on expression levels. Their basic characteristics are shown in Table S5. The high TNFSF15 expression group exhibited significantly poorer histological differentiation than did the low TNFSF15 expression group (*p* = 0.010). Furthermore, if the patients were divided into well and moderate‐to‐poor differentiation groups according to their histologic differentiation, the patients with moderate‐to‐poor histological differentiation demonstrated higher TNFSF15 expression than did those with well‐differentiated tumours, both in all the patients with HNSCC and the human papillomavirous (HPV)‐negative subgroup (mean ± SD for TNFSF15 expression, moderate‐to‐poor vs. well, 19.61 ± 29.61 vs. 11.15 ± 10.48 for all the patients with HNSCC, *p* = 0.0263 and 16.83 ± 26.67 vs. 10.79 ± 10.43 for the HPV‐negative group, *p* = 0.0896, respectively; Figure 2).

**FIGURE 3:**
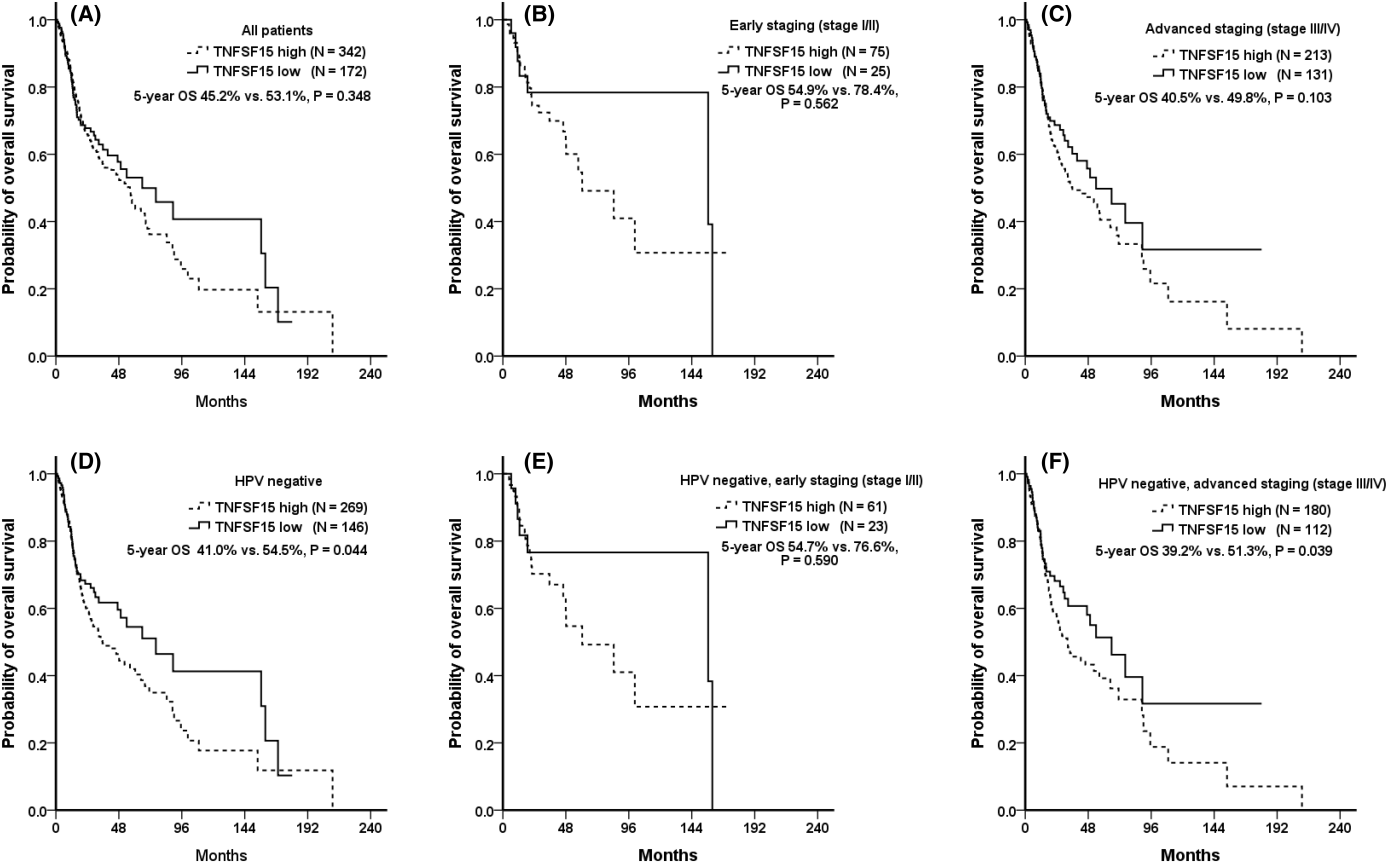
TNFSF15 expression and overall survival In TCGA database. In TCGA database, TNFSF15 expression and overall survival in (A) All OCSCC, (B) Early staging, (C) advanced staging, (D) HPV negative, (E) HPV negative and Early staging, and (F) HPV negative and advanced staging population

Among all the patients, the 5‐year OS of the high and low TNFSF15 expression groups was 45.2% and 53.1%, respectively (*p* = 0.348; early staging, 54.9% vs. 78.4%, *p* = 0.562 and advanced staging, 40.5% vs. 49.8%, *p* = 0.103, respectively; Figure 3A–C). For the HPV‐negative subgroup, the 5‐year OS of the high and low TNFSF15 expression groups was 41.0% and 54.5%, respectively (*p* = 0.044; early staging, 54.7% vs. 76.6%, *p* = 0.590 and advanced staging, 39.2% vs. 51.3%, *p* = 0.039, respectively; Figure 3D–F). Those with high TNFSF15 expression, which might be associated with altered *TNFSF15* correlated to advanced histological differentiation, had poorer OS than did those with low TNFSF15 expression, especially the HPV‐negative and advanced staging populations.

## 4 DISCUSSION

A total of 2523 participants (1324 patients with OCSCC and 1199 healthy controls) were enrolled in this study. The *TNFSF15* SNVs did not affect the development of OCSCC. However, the patients with OCSCC with altered *TNFSF15* SNVs exhibited poorer histological differentiation than did those with wild‐type alleles among all the patients and betel quid chewers. In the univariate logistic regression analysis, the altered *TNFSF15* SNVs were significant for moderate‐to‐poor differentiation. We analysed the published bioinformatics databases and determined that the altered SNVs had lower expression levels in whole blood but higher expression levels in the upper aerodigestive (oesophagus) mucosa compared with the expression levels of wild‐type alleles. The TCGA database indicated that those with high TNFSF15 expression, which might be associated with allelic variations and advanced histological differentiation, had poorer OS than did those with low TNFSF15 expression, especially the HPV‐negative and advanced staging populations. Future studies are warranted to verify these results.

The strengths of this study are as follows. First, in this large case–control study, a total of 2523 participants were enrolled. In addition, although *TNFSF15* coactivates T cells and is associated with the development of inflammatory diseases,^12^, ^15^, ^16^, ^17^ interactions between *TNFSF15* SNVs and OCSCC, which are related to inflammatory reactions caused by personal health habits, were unknown. This study aimed to fill these gaps; however, future advanced in vitro studies are needed. Third, in previous studies focusing on IBD, *TNFSF15* SNVs were especially relevant to the Asian population.^15^, ^16^ Some personal habits are unique to the Asian population, such as betel quid chewing, which may result in HPV‐negative OCSCC. Thus, the effects of *TNFSF15* SNVs on the Asian population are worthy of attention. Finally, our results were validated using published bioinformatic databases.

The interactions of *TNFSF15* SNVs with inflammatory disorders, such as IBD, have been widely studied. Zhang et al. performed a meta‐analysis and reported that *TNFSF15* SNVs were significantly associated with the development of Crohn's disease and ulcerative colitis, especially in the Asian population.^15^ Park et al. indicated that genetic heterogeneities were different between the Asian and Western populations and that *TNFSF15* SNVs, such as rs6478108 and rs6478109, significantly contributed to the risk of IBD.^16^ Gao et al. demonstrated that *TNFSF15* rs7848647 and rs6478109 were more likely to cause small‐cell lung cancer (rs7848647, OR [95% CI] = 1.84 [1.13–2.99] and rs6478109, 2.44 [1.46–4.06]).^13^ Slebioda et al. reported that *TNFSF15* encodes TL1A. Altered *TNFSF15* rs6478108 and rs6478109 were associated with an increased expression of TL1A, and the patients with higher TL1A expression had poorer survival than did those with lower TL1A expression. The expression of TL1A was determined to be an independent factor for overall survival in Cox regression analysis.^11^, ^18^ These results indirectly emphasize the significance of *TNFSF15* SNVs in colorectal cancer. In our study, although altered *TNFSF15* SNVs did not affect the development of OCSCC, altered *TNFSF15* SNVs were significantly associated with poorer histological differentiation than were the wild‐type alleles. The published databases indicated that the upper aerodigestive (oesophagus) mucosa with altered *TNFSF15* exhibited higher TNFSF15 expression than did that with wild‐type *TNFSF15*. The patients with higher TNFSF15 expression had poorer prognosis than did those with lower TNFSF15 expression, especially HPV‐negative and advanced staging populations.

The TNF superfamily has several ligand–receptor pairs and the pair TNFSF15–DR3 is one of them.^10^ TNFSF15, induced by TNF‐α and interleukin (IL)‐1α, is the ligand expressed on antigen‐presenting cells, CD4+/CD8+ T cells, and endothelial cells. Activation of TNF ligands can promote the secretion of proinflammatory cytokines, such as TNF, IL‐1, IL‐6 and IL‐12, and lead to cellular proliferation. In addition, the DR3 receptor is expressed on T cells, natural killer (NK) cells and NK T cells. Nuclear factor‐κB (NF‐κB) is the main downstream signal observed after triggering TNF receptors, and it contributes to the production of cytokines, such as IL‐2, IL‐4, IL‐5 and interferon‐γ.^10^, ^52^ Several diseases are associated with the TNFSF15–DR3 pair, including autoimmune diseases and IBD.^53^, ^54^ Several studies have reported that the downstream cytokines of the TNFSF15–DR3 pair, such as IL‐6, IL‐8 and TNF‐α, may serve as biomarkers for the early diagnosis and prognosis of OCSCC.^55^, ^56^ Some of these cytokines were correlated with histological grading.^57^ However, the interaction between TNFSF15 and OCSCC has rarely been discussed, especially for betel quid chewers.

In our study, *TNFSF15* SNVs were independent to moderate‐to‐poor histologic differentiation in univariant Cox regression analysis. The mechanism between TNFSF15 expression and histologic grade in OCSCC was unclear. In Parr et al. study, TNFSF15 expression was positively correlated to moderate‐to‐poor histologic grade.^58^ In addition, higher TNFSF15 expression was corresponding to higher E‐cadherin expression,^59^ a biomarker of epithelial‐mesenchymal transition that the patients with higher E‐cadherin expression were indirect with poorly histologic grade.^60^, ^61^ And future studies were warranted.

This study has several limitations. Although more than 2000 participants were retrospectively enrolled in this study, a validation cohort was still required. In addition, in our study, DNA was extracted from different specimens to sequence *TNFSF15* SNVs, including the whole blood of all the enrolled participants and the tumour tissue specimens from the TCGA database. Some studies have extracted predictive cytokines from saliva samples.^55^, ^56^ Based on Figure S1, the interaction between *TNFSF15* SNVs and expression might vary among different specimens. In upper aerodigestive (oesophagus) mucosa, TNFSF15 expressions of altered *TNFSF15* alleles were higher than those of wild‐type. Advanced in vitro and in vivo validations for specimens are needed. Third, the function of individual *TNFSF15* SNVs might differ, and some SNVs were reported to protect against IBD.^62^ Thus, functional experiments for individual SNVs should be conducted. Finally, because of delinking and anonymity, we could not retrospectively review the clinical outcomes of the enrolled participants. Advanced studies examining the functions of individual SNVs and participants' clinical outcomes should be conducted in the future.

In conclusion, *TNFSF15* SNVs did not affect the development of OCSCC. However, mutant *TNFSF15* SNVs were associated with poorer histological differentiation. Validated published databases indicated that altered *TNFSF15* SNVs resulted in higher TNFSF15 expression in the upper aerodigestive (oesophagus) mucosa than did the wild‐type alleles. The patients with higher TNFSF15 expression in the upper aerodigestive (oesophagus) mucosa had poorer OS than did those with lower TNFSF15 expression, especially HPV‐negative and advanced staging populations. Related in vitro and in vivo studies are warranted in the future.

## AUTHOR CONTRIBUTIONS

**Hsueh‐Ju Lu:** Conceptualization (equal); writing – original draft (equal); writing – review and editing (equal). **Chun‐Yi Chuang:** Resources (equal). **Chun‐Wen Su:** Methodology (equal). **Mu‐Kuan Chen:** Resources (equal). **Wei‐En Yang:** Methodology (equal). **Chia‐Ming Yeh:** Methodology (equal). **Chih‐Hsin Tang:** Methodology (equal). **Chiao‐Wen Lin:** Conceptualization (equal); writing – original draft (equal); writing – review and editing (equal). **Shun‐Fa Yang:** Conceptualization (equal); writing – original draft (equal); writing – review and editing (equal).

## FUNDING INFORMATION

This study was supported by research grants from the Chung Shan Medical University Hospital, Taiwan (CSH‐2022‐C‐014) to Hsueh‐Ju Lu.

## CONFLICT OF INTEREST

The authors declare that there is no conflict of interest.

## Supporting information


**Appendix S1:** Supporting information

## Data Availability

The data used to support the findings of the present study are available from the corresponding author upon request.
