# Supplementary material for: Role of TNFSF15 variants in oral cancer development and clinicopathologic characteristics
Source: J Cell Mol Med. 2022 Oct 13;26(21):5452–62. doi: 10.1111/jcmm.17569 (PMC9639028; doi:10.1111/jcmm.17569)
Supplement: Supplementary file 1 — Appendix S1: Supporting information [file JCMM-26-5452-s001.pdf]

Table S1. Detailed information of *TNFSF15* SNPs from dbSNP

dbSNP	rs3810936	rs6478108	rs6478109
Organism	*Homo sapiens*	*Homo sapiens*	*Homo sapiens*
Position[1]	Chr9:114790605	Chr9:114796423	Chr9:114806486
Nucleotide change	T>C	C>T	A>G
Variation type	SNV	SNV	SNV
Minor allele frequency[2]	C=0.5069	T=0.5099	G=0.5122
Gene consequence	synonymous variant	intron variant	2KB upstream variant

[1] GRCh38.p13

[2] Minor allele frequency using the East Asian population from 1000 Genomes

Table S2. The distributions of demographical characteristics of *TNFSF15* allele mutation in OCSCC patients with cigarette smoking (N = 1115)

	**rs3810936**			**rs6478108**			**rs6478109**		
Variable	TC+CC (N=784)	TT (N=331)	*P* value	CT+TT (N=815)	CC (N=300)	*P* value	AG+GG (N=823)	AA (N=292)	*P* value
**Age >= 55**	403 (51.4)	164 (49.5)	0.308	425 (52.1)	142 (47.3)	0.087	432 (52.5)	135 (46.2)	0.038
**Personal history**									
alcohol drinking	402 (51.3)	181 (54.7)	0.165	422 (51.8)	161 (53.7)	0.312	425 (51.6)	158 (54.1)	0.256
betel quid chewing	646 (82.4)	281 (84.9)	0.177	673 (82.6)	254 (84.7)	0.232	679 (82.5)	248 (84.9)	0.195
**Pathologic staging**			0.136			0.069			0.030
Stage I+II	361 (46.0)	165 (49.8)		373 (45.8)	153 (51.0)		374 (45.4)	150 (52.1)	
Stage III+IV	423 (54.0)	166 (50.2)		442 (54.2)	147 (49.0)		449 (54.6)	140 (47.9)	
**Pathologic T staging**			0.159			0.057			0.069
T1/2	399 (50.9)	180 (54.4)		411 (50.4)	168 (56.0)		416 (50.5)	163 (55.8)	
T3/4	385 (49.1)	151 (45.6)		404 (49.6)	132 (44.0)		407 (49.5)	129 (44.2)	
**Pathologic N staging**			0.166			0.113			0.045
N0	510 (65.1)	226 (68.3)		529 (64.9)	207 (69.0)		531 (64.5)	205 (70.2)	
N+	274 (34.9)	105 (31.7)		286 (35.1)	93 (31.0)		292 (35.5)	87 (29.8)	
**Metastasis**			0.529			0.547			0.567
M0	778 (99.2)	328 (99.1)		808 (99.1)	298 (99.3)		816 (99.1)	290 (99.3)	
M1	6 (0.8)	6 (0.8)		7 (0.9)	2(0.7)		7 (0.9)	2 (0.7)	
**Cell differentiated grade**			0.002			0.007			0.004
Well	100 (12.8)	65 (19.6)		107 (13.1)	58(19.3)		107 (13.0)	58 (19.9)	
Moderate or poor	684 (87.2)	266 (80.4)		708 (86.9)	242(80.7)		716 (87.0)	234 (80.1)	

Table S3. The distributions of demographical characteristics of *TNFSF15* allele mutation in OCSCC patients with alcohol drinking (N = 625)

	**rs3810936**			**rs6478108**			**rs6478109**		
Variable	TC+CC (N=427)	TT (N=198)	*P* value	CT+TT (N=449)	CC (N=176)	*P* value	AG+GG (N=452)	AA (N=173)	*P* value
**Age >= 55**	209 (48.9)	77 (38.9)	0.012	214 (47.7)	72 (40.9)	0.075	217 (48.0)	69 (39.9)	0.041
**Personal history**									
cigarette smoking	409 (94.1)	181 (91.4)	0.137	422 (94.0)	161 (91.5)	0.171	425 (94.0)	158 (91.3)	0.152
betel quid chewing	377 (88.3)	174 (87.9)	0.489	394 (87.8)	157 (89.2)	0.361	396 (87.6)	155 (89.6)	0.296
**Pathologic staging**			0.054			0.044			0.037
Stage I+II	189 (44.3)	102 (51.5)		199 (44.3)	92 (52.3)		200 (44.2)	91 (52.6)	
Stage III+IV	238 (55.7)	96 (48.5)		250 (55.7)	84 (47.7)		252 (55.8)	82 (47.4)	
**Pathologic T staging**			0.140			0.149			0.214
T1/2	218 (51.1)	111 (56.1)		230 (51.2)	99 (56.3)		233 (51.5)	96 (55.5)	
T3/4	209 (48.9)	87 (43.9)		219 (48.8)	77 (43.8)		219 (48.5)	77 (44.5)	
**Pathologic N staging**			0.290			0.171			0.173
N0	269 (63.0)	130 (65.7)		281 (62.6)	118 (67.0)		283 (62.6)	116 (67.1)	
N+	158 (37.0)	68 (34.3)		168 (37.4)	58 (33.0)		169 (37.4)	57 (32.9)	
**Metastasis**			0.148			0.311			0.300
M0	424 (99.3)	194 (98.0)		445 (99.1)	173 (98.3)		448 (99.1)	170 (98.3)	
M1	3 (0.7)	3 (2.0)		4 (0.9)	3(1.7)		4 (0.9)	3 (1.7)	
**Cell differentiated grade**			<0.001			<0.001			<0.001
Well	45 (10.5)	43 (21.7)		48 (10.7)	40 (22.7)		48 (10.6)	40 (23.1)	
Moderate or poor	382 (89.5)	155 (78.3)		401 (89.3)	136 (77.3)		404 (89.4)	133 (76.9)	

Table S4. The distributions of demographical characteristics of *TNFSF15* allele mutation in OCSCC patients with betel quid chewing (N = 989).

	**rs3810936**			**rs6478108**			**rs6478109**		
Variable	TC+CC (N=690)	TT (N=299)	*P* value	CT+TT (N=716)	CC (N=273)	*P* value	AG+GG (N=722)	AA (N=267)	*P* value
**Age >= 55**	356 (51.6)	144 (48.2)	0.178	367 (51.3)	133 (48.7)	0.260	373 (51.7)	127 (47.6)	0.142
**Personal history**									
cigarette smoking	646 (93.6)	281 (94.0)	0.479	673 (94.0)	254 (93.0)	0.336	679 (94.0)	248 (92.9)	0.296
alcohol drinking	377 (54.6)	174 (58.2)	0.167	394 (55.0)	157 (57.5)	0.264	396 (54.8)	155 (58.1)	0.204
**Pathologic staging**			0.162			0.051			0.020
Stage I+II	321 (46.5)	150 (50.2)		329 (45.9)	142 (52.0)		329 (45.6)	142 (53.2)	
Stage III+IV	369 (53.5)	149 (49.8)		387 (54.1)	131 (48.0)		393 (54.4)	125 (46.8)	
**Pathologic T staging**			0.222			0.046			0.059
T1/2	347 (50.3)	159 (53.2)		354 (49.4)	152 (55.7)		358 (49.6)	148 (55.4)	
T3/4	343 (49.7)	140 (46.8)		362 (50.6)	121 (44.3)		364 (50.4)	119 (44.6)	
**Pathologic N staging**			0.281			0.104			0.042
N0	456 (66.1)	204 (68.2)		469 (65.5)	191 (70.0)		470 (65.1)	190 (71.2)	
N+	234 (33.9)	95 (31.8)		247 (34.5)	82 (30.0)		252 (34.9)	77 (28.8)	
**Metastasis**			0.642			0.618			0.604
M0	685 (99.3)	297 (99.3)		711 (99.3)	271 (99.3)		717 (99.3)	265 (99.3)	
M1	5 (0.7)	2 (0.7)		5 (0.7)	2(0.7)		5 (0.7)	2 (0.7)	
**Cell differentiated grade**			0.002			0.003			0.001
Well	88 (12.8)	61 (20.4)		93 (13.0)	56 (20.5)		93 (12.9)	56 (21.0)	
Moderate or poor	602 (87.2)	238 (79.6)		623 (87.0)	217(79.5)		629 (87.1)	211 (79.0)	

Table S5. Basic characteristics of high- and low- TNFSF15 expression patients diagnosed HNSCC from the TCGA database

Variable	High TNFSF15 (N=343)	Low TNFSF15 (N=172)	*P* value
Age (yrs)			*0.333*
≧55	247(72.0)	120(69.8)	
<55	96(28.0)	52(30.2)	
Gender			0.233
Male	257(74.9)	123(71.5)	
Female	86(25.1)	49(28.5)	
Pathologic staging			0.006
I+II	76(22.2)	25(14.5)	
III+IV	213(62.1)	131(76.2)	
Unknown	54(15.7)	16(9.3)	
Pathologic T staging			0.007
T1+T2	134(39.1)	49(28.5)	
T3+T4	163(47.5)	107(62.2)	
Unknown	46(13.4)	16(9.3)	
Pathologic N staging			0.123
N0	116(33.8)	58(33.7)	
N+	153(44.6)	89(51.7)	
Unknown	74(21.6)	25(14.5)	
Pathologic M staging			0.075
M0	113(32.9)	70(40.7)	
M1	0(0.0)	1(0.6)	
Unknown	230(67.1)	101(58.7)	
Histological differentiation			0.010
Well	36(10.5)	26(15.1)	
Moderate	198(57.7)	103(59.9)	
Poor	84(24.5)	39(22.7)	
Undifferentiated	7(2.0)	0(0.0)	
Unknown	18(3.5)	4(2.3)	
HPV statue			0.215
Positive	53(15.5)	19(11.0)	
Negative	269(78.4)	146(84.9)	
Unknown	21(6.1)	7(4.1)	

Figure S1

(A) rs3810936

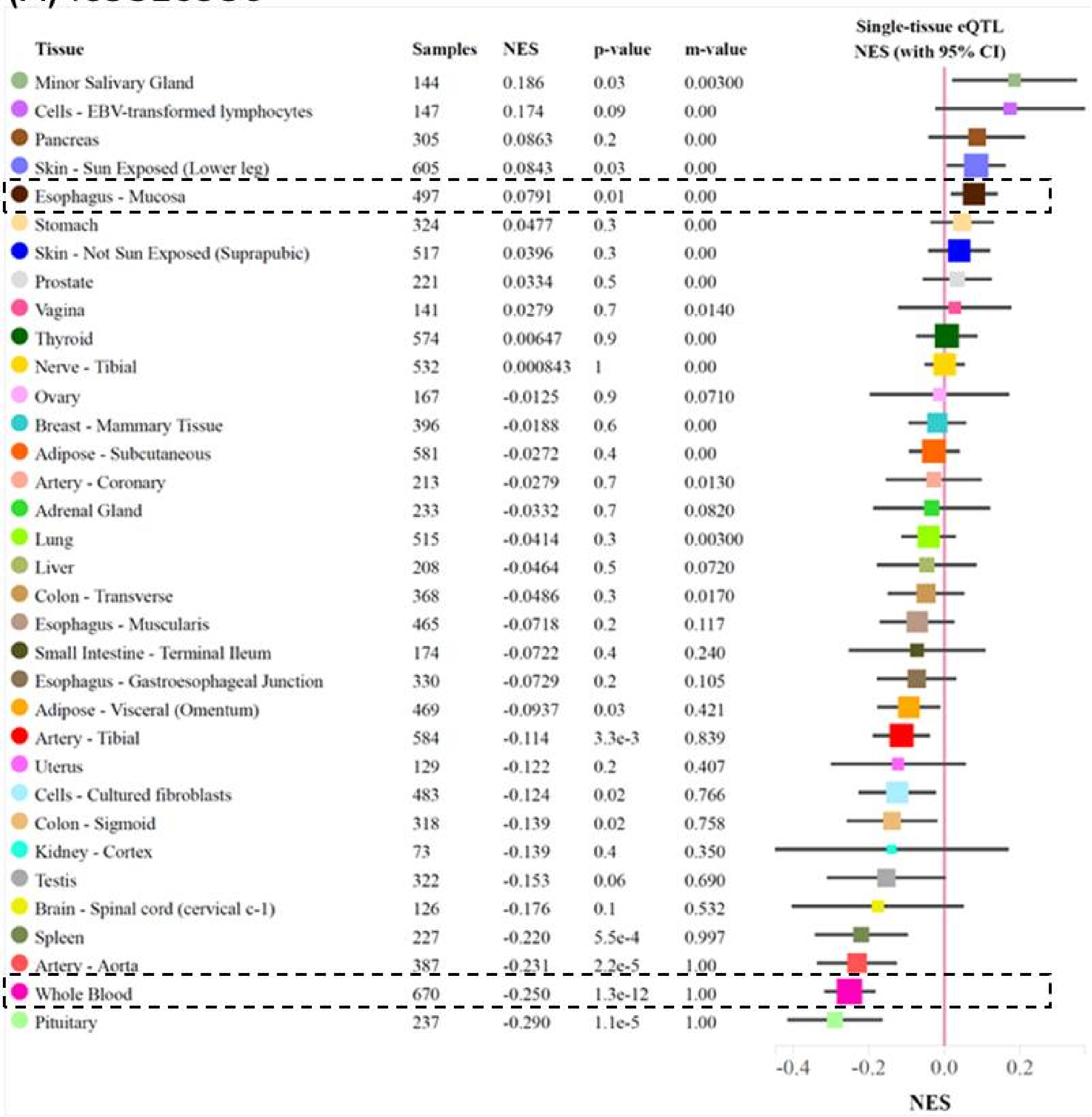

(B) rs6478108

Tissue	Samples	NES	p-value	m-value
Minor Salivary Gland	144	0.126	0.1	0.00
Esophagus - Mucosa	497	0.100	8.7e-4	0.00
Pancreas	305	0.0919	0.1	0.00
Cells - EBV-transformed lymphocytes	147	0.0816	0.4	0.00
Stomach	324	0.0653	0.1	0.00
Skin - Sun Exposed (Lower leg)	605	0.0636	0.1	0.00
Prostate	221	0.0469	0.3	0.00
Vagina	141	0.0234	0.8	0.0120
Skin - Not Sun Exposed (Suprapubic)	517	0.0228	0.6	0.00
Nerve - Tibial	532	0.0116	0.7	0.00
Colon - Transverse	368	0.00363	0.9	0.00
Breast - Mammary Tissue	396	-0.000722	1	0.00
Thyroid	574	-0.00241	1	0.00
Artery - Coronary	213	-0.00533	0.9	0.0120
Lung	515	-0.00828	0.8	0.00
Ovary	167	-0.0310	0.7	0.0460
Esophagus - Muscularis	465	-0.0312	0.5	0.00
Adipose - Subcutaneous	581	-0.0407	0.2	0.00
Esophagus - Gastroesophageal Junction	330	-0.0444	0.4	0.00
Liver	208	-0.0568	0.4	0.0600
Adrenal Gland	233	-0.0817	0.3	0.133
Uterus	129	-0.0840	0.4	0.184
Small Intestine - Terminal Ileum	174	-0.105	0.2	0.137
Colon - Sigmoid	318	-0.111	0.08	0.239
Adipose - Visceral (Omentum)	469	-0.112	0.006	0.451
Artery - Tibial	584	-0.113	2.7e-3	0.516
Cells - Cultured fibroblasts	483	-0.127	0.01	0.583
Testis	322	-0.128	0.1	0.376
Brain - Spinal cord (cervical c-1)	126	-0.151	0.1	0.401
Spleen	227	-0.176	3.5e-3	0.902
Kidney - Cortex	73	-0.177	0.2	0.338
Pituitary	237	-0.225	5.3e-4	0.997
Artery - Aorta	387	-0.245	2.7e-6	1.00
Whole Blood	670	-0.293	3.5e-18	1.00

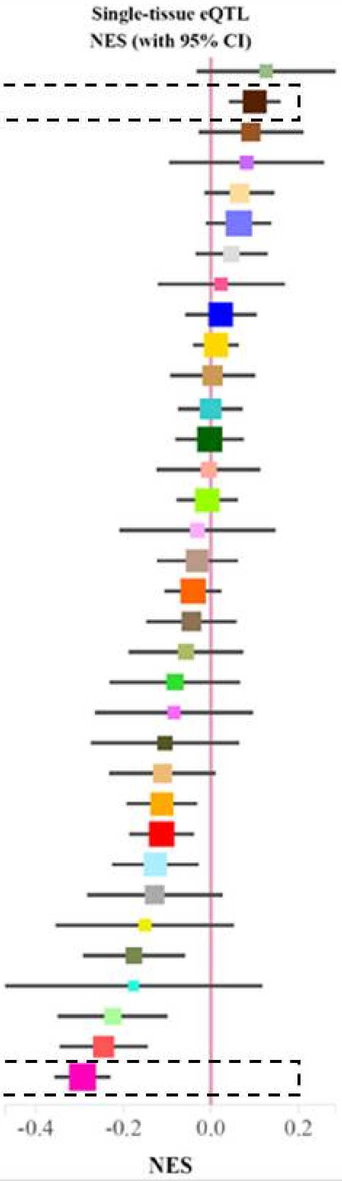

(C) rs6478109

Tissue	Samples	NES	p-value	m-value
Minor Salivary Gland	144	0.135	0.09	0.00
Esophagus - Mucosa	497	0.0991	1.1e-3	0.00
Cells - EBV-transformed lymphocytes	147	0.0949	0.3	0.00400
Skin - Sun Exposed (Lower leg)	605	0.0752	0.06	0.00
Stomach	324	0.0590	0.2	0.00
Pancreas	305	0.0553	0.4	0.00
Prostate	221	0.0372	0.4	0.00
Vagina	141	0.0246	0.7	0.0200
Skin - Not Sun Exposed (Suprapubic)	517	0.0165	0.7	0.00
Ovary	167	0.0141	0.9	0.0640
Nerve - Tibial	532	0.00730	0.8	0.00
Artery - Coronary	213	0.00398	0.9	0.00700
Lung	515	0.00196	1	0.00
Thyroid	574	-0.0000474	1	0.00
Breast - Mammary Tissue	396	-0.0123	0.7	0.00
Adipose - Subcutaneous	581	-0.0192	0.6	0.00
Colon - Transverse	368	-0.0262	0.6	0.0220
Esophagus - Muscularis	465	-0.0438	0.4	0.00500
Liver	208	-0.0563	0.4	0.0830
Esophagus - Gastroesophageal Junction	330	-0.0646	0.2	0.0650
Uterus	129	-0.0982	0.3	0.288
Adrenal Gland	233	-0.102	0.2	0.318
Adipose - Visceral (Omentum)	469	-0.112	0.007	0.632
Artery - Tibial	584	-0.117	2.5e-3	0.793
Cells - Cultured fibroblasts	483	-0.125	0.02	0.711
Small Intestine - Terminal Ileum	174	-0.127	0.2	0.397
Colon - Sigmoid	318	-0.161	0.01	0.929
Testis	322	-0.163	0.04	0.710
Brain - Spinal cord (cervical c-1)	126	-0.167	0.1	0.658
Kidney - Cortex	73	-0.174	0.3	0.458
Spleen	227	-0.179	4.0e-3	0.952
Pituitary	237	-0.241	4.3e-4	1.00
Artery - Aorta	387	-0.257	1.9e-6	1.00
Whole Blood	670	-0.286	1.3e-16	1.00

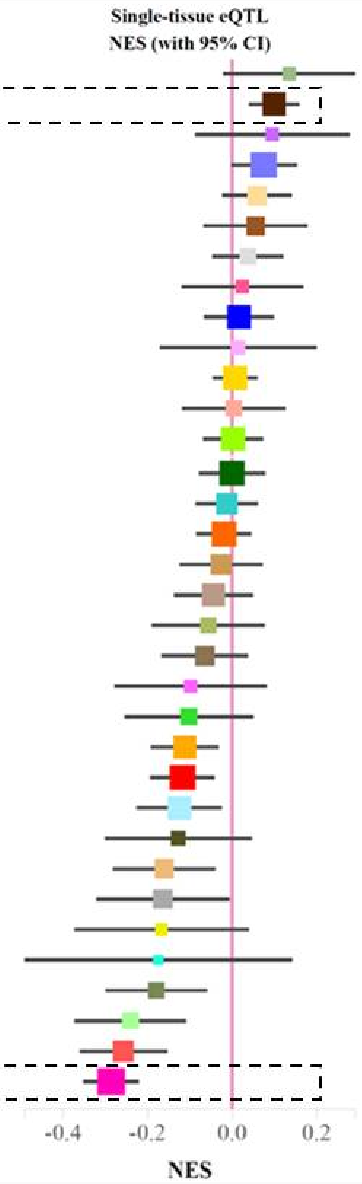

Figure S1. TNFSF15 expression in different tissue types, presented based on TNFSF15 allele mutation. The data of single-tissue QTL normalized effect size and P value in (A) *TNFSF15* rs3810936 (B) *TNFSF15* rs6478108 and (C) *TNFSF15* rs6478109.
